# Effect of cognitive behavioral intervention on electroencephalographic band powers of children with learning difficulty under eyes-closed and eyes-open conditions

**DOI:** 10.1590/0004-282X-ANP-2020-0503

**Published:** 2022-02-28

**Authors:** Pratima Kaushik, Samanta Puspak Kumar Jena

**Affiliations:** 1 Jesus and Mary College, Department of Psychology, University of Delhi, New Delhi, India. Jesus and Mary College Department of Psychology University of Delhi New Delhi India; 2 University of Delhi, South Campus Unit, Department of Applied Psychology, New Delhi, India. University of Delhi South Campus Unit Department of Applied Psychology New Delhi India

**Keywords:** Electroencephalography, Learning, Cognitive Behavioral Therapy, Cognition, Resonance Frequency Analysis, Electroencefalografía, Aprendizaje, Terapia Cognitivo-Conductual, Cognición, Análisis de Frecuencia de Resonancia

## Abstract

**Background:**

Electroencephalography (EEG) plays an important role in assessing children with learning difficulties or related behavioral issues. Understanding EEG alterations in students with learning difficulties is crucial for evaluating cognitive functioning.

**Objective:**

The first aim was to examine the effects of the Program for Enhancing Academic and Behavioral Learning Skills (PEABLS), a cognitive-behavioral intervention on absolute and relative EEG band powers under eyes-closed and eyes-open conditions. Another aim was to examine the relationship between relative band powers of EEG waveforms through specific cognitive measurements like IQ, working memory and BGT for perceptual motor skills and organization.

**Methods:**

This study had a quasi-experimental pre-test post-test research design and involved a group of 50 students with learning problems. PEABLS, an accessible school-based intervention, was offered to academically low-performing students. EEG recordings were conducted before and after the intervention on prefrontal (FP1 FP2), temporal (T3 T4) and occipital (O1 O2) scalp locations. The data acquired were processed using MATLAB to find the absolute and relative band powers of waveforms.

**Results:**

Paired t tests on the recorded EEG data suggested that significant improvements in absolute and relative power values of waveforms were achieved, post-intervention. There were significant increases in relative alpha power values in the prefrontal and temporal regions under both eyes-closed and eyes-open conditions and significant increases in relative theta and delta power in the prefrontal and temporal regions. Pearson's correlation suggested that there was a significant relationship between relative alpha and beta power values in the prefrontal and occipital regions, through the cognitive measurements.

**Conclusion:**

PEABLS was significative in bringing changes to EEG band powers.

## RESUMEN

**Antecedentes:**

comprender las alteraciones del electroencefalograma en estudiantes con dificultades de aprendizaje es fundamental para evaluar el funcionamiento cognitivo.

**Objetivo:**

El primer objetivo fue examinar los efectos del Programa para mejorar las habilidades de aprendizaje académico y conductual (PEABLS), una intervención cognitivo-conductual sobre los poderes absolutos y relativos de la banda de EEG en condiciones de ojos cerrados y abiertos. Otro objetivo fue examinar la relación entre los poderes de banda relativos de las formas de onda del EEG a través de medidas cognitivas específicas como el coeficiente intelectual, la memoria de trabajo y BGT para las habilidades motoras perceptivas y la organización.

**Métodos:**

Este estudio tuvo un diseño de investigación cuasi-experimental pre-test post-test e involucró a un grupo de 50 estudiantes con problemas de aprendizaje. PEABLS, una intervención accesible basada en la escuela, se ofreció a estudiantes de bajo rendimiento académico. Los registros de EEG se realizaron antes y después de la intervención en las localizaciones del cuero cabelludo prefrontal (FP1 FP2), temporal (T3 T4) y occipital (O1 O2). Los datos adquiridos se procesaron utilizando MATLAB para encontrar las potencias de banda absolutas y relativas de las formas de onda.

**Resultados:**

las pruebas t pareadas en los datos de EEG registrados sugirieron que se lograron mejoras significativas en los valores de potencia absoluta y relativa de las formas de onda, después de la intervención. Hubo aumentos significativos en los valores de potencia alfa relativa en las regiones prefrontal y temporal y en la potencia relativa theta y delta en las regiones prefrontal y temporal. La correlación de Pearson sugirió que había una relación significativa entre los valores de potencia relativa alfa y beta a través de las mediciones cognitivas.

**Conclusión:**

PEABLS fue significativo al traer cambios a los poderes de la banda de EEG.

## INTRODUCTION

Students with learning difficulty are identified as having difficulties in learning one or more academic skills. Their academic grades lie below the anticipated scores for students of the same age, class and school environment. Low socioeconomic status and unfavorable circumstances negatively impact their cognitive and academic performance, which leads to repeated failures and causes a higher risk of developing learning difficulty. Although these students may score moderate to above average on IQ tests, they underperform scholastically due to a conflux of pedagogical reasons. This may include family psychopathology, sociocultural environment with adverse conditions, lack of opportunity to learn cognitive skills, sensory deprivation, emotional causes such as demotivation and migration (leading to sociolinguistic interaction deprivation), poor quality of education and impoverished socioeconomic and cultural conditions. Such conditions strongly and negatively influence their cognitive development and academic performance^1^^,^^2^. 

Investigations into the neurocognitive functioning of socially disadvantaged children have suggested that these children present compromised psychosocial functioning, psychopathology, brain dysfunction and cognitive deficits, including impaired executive functioning, attention, processing speed, language, memory and social skills^3^^,^^4^. Unfortunately, the problems faced by these children often go unrecognized since these fail to be classified under the definitive diagnostic category of developmental disorder, given that there is no organic deficiency and the cause is external to the individual^5^. Learning difficulty has also been termed school difficulty ^6^, scholastic backwardness^7^ and poor school performance ^8^ in several studies. 

Quantitative electroencephalogram (qEEG) studies have revealed alterations in the absolute and relative band powers of children with learning disabilities, compared with their counterparts without learning disabilities. Increased absolute power in the delta and theta bands ^9^^-^^11^ and reduced alpha and beta activity^12^^,^^13^ have been observed. Some studies have reported a positive connection between severity of reading/writing disabilities and delta activity, particularly in the frontal and temporal regions^12^^,^^14^. Individuals with mild disabilities had high absolute and relative theta activity and low relative alpha activity. 

The abnormalities among these children tend to decrease over time, thus suggesting that maturity factors are important^14^. Slow EEG activity has been found to be suggestive of learning disability^15^. However, other studies have not revealed any significant differences between healthy controls and dyslexic children (e.g.^16^^-^^19^). Children with attention-deficit/hyperactivity disorder (ADHD) have been shown to have slower beta activity^17^^-^^19^. 

Studies have also evaluated the relationship between qEEG variables and IQ measurements^20^^-^^22^. Children with learning disability not otherwise specified (LD-NOS) were found to have differences in EEG activity under eyes-open conditions in the left temporal cortex at delta, theta, alpha and beta frequencies, and alpha activity under eyes-closed conditions. An increased theta/alpha ratio in the frontal regions was also found^23^. In another study, it was stated that the slow learner group had marginally more significant low beta at the left than at the right temporal site^24^.

Children show improvement in learning academic, cognitive and psychosocial skills after receiving focused training. It has become crucial to understand the impact of cognitive-behavioral interventions on EEG characterizations of such children. The Program for Enhancing Academic and Behavioral Learning Skills (PEABLS) is an intervention based on a cognitive-behavioral technique that aims to "normalize" deviant brain activity, thereby resulting in improved behavioral and cognitive performance. 

The present study was designed to carry out EEG assessments before and after introducing training sessions based on the Program for Enhancing Academic and Behavioral Learning Skills (PEABLS). This program focuses on training of self-regulation and resiliency skills that help improve the academic performance of students with learning difficulties. The aim of this study was to identify differences in resting-state EEGs under eyes-closed and eyes-open conditions among such children. We proposed to analyze EEG activity in children facing problems in learning and to evaluate the relationship of EEGs to academic, cognitive and behavioral measurements. 

## METHODS

### Sampling

This study was quasi-experimental with a pre-test, post-test experimental-group research design. Approval from the authorities concerned was obtained before approaching the Principal of a primary school. Students were identified based on their academic records over the last two years, the teacher's feedback and the parents’ reports. The students included in this study had the following characteristics: all of them were living in urban slums; their ages ranged from 8 to 12 years (3^rd^ to 7^th^ grade); and they had average IQ scores, had failed in class for two consecutive years, had low classroom participation and co-curricular activity and had behavioral issues.

However, students with any physical disability, intellectual disability, sensory impairments or any other developmental disorder were excluded from the study. The teachers were briefed about the project, and a list of fifty students with learning problems was identified. None of these students withdrew from the study after the intervention started. Their parents were oriented about the intervention at the annual parent-teacher meeting. Informed consent was obtained from them for the participation of these students in this study. To select the age group for the present study, previous EEG research was used as the reference point^22^^,^^25^. 

This study was approved by the Indian Council for Social Science Research and the Department of Psychology, University of Delhi. The ethical principles for medical research involving human subjects established through the Declaration of Helsinki were followed. 

The student selection, assessment and intervention were conducted under the supervision of a clinical psychologist, who was also the co-author of this study. The intervention was provided in two locations: school premises and the psychophysiology laboratory of the Department of Applied Psychology, University of Delhi. Refer to Table 1 for details of the student participants.

**Table 1.** Details of the participants.

Variables	N	Minimum	Maximum	Mean	SD
Age	50	8	12	10.38	1.398
Grade	50	3	7	4.78	1.329
Aggregate marks in % (pre-intervention)	50	23	54	37.90	7.005
Aggregate marks in % (post-intervention)	50	35	62	47.78	6.81
Body mass index	50	11	21	13.88	2.017

SD: standard deviation.

The study proceeded in three phases: phase I -pre-intervention assessment; phase II -intervention; and phase III -post-intervention assessment. 

During phase I, all students identified were screened for their IQ scores by means of Raven's Colored Progressive Matrices (RCPM) and scores in the Diagnostic Test of Learning Disability (DTLD). The students who obtained IQ ≥ 50^th^ percentile in the RCPM and a score of ≥ 40% in the DTLD were further subjected to the Digit Span Test, forward and backward, which is a subtest of Malin's Intelligence Test for Indian Children, to assess their working memory status. The Bender Gestalt Test (BGT) to assess visual-motor functioning and EEGs was also recorded. The detailed process of obtaining qEEG data is discussed in the next section of this article. 

During phase II, the Program for Enhancing Academic and Behavioral Learning Skills (PEABLS), which is a cognitive-behavioral intervention, was administered to 50 participants. The pre-intervention assessments were done on these students after they had satisfied the inclusion and exclusion criteria. PEABLS was developed to address the needs of schoolchildren with learning difficulty, to cater for their low academic performance and behavioral and emotional issues through providing an accessible school-based intervention. It consists of three components: (i) cognitive assessment; (ii) problem-solving and decision-making assessment; and (iii) academic remediation. The PEABLS intervention was provided for two months (16 sessions), which helped the students to identify their negative beliefs and develop cognitive flexibility when confronted with negative thoughts. The students were taught to resolve interpersonal conflicts, develop assertive communication and control aggressive and impulsive spells. Individualized educational remediation helped to fill conceptual deficits^26^.

In phase III, post-intervention assessments were conducted in order to obtain data on cognitive and EEG measurements. 

### Measurements

The students were assessed twice, using two types of measurement: EEGs and the cognitive measurements described below. 

### Cognitive measurements

- (i) The Diagnostic Test of Learning Disability (DTLD)^27^ is a screening tool for measuring students' visual and auditory perception and cognitive functioning.
- (ii) Raven's Colored Progressive Matrices (RCPM)^28^ is the standardized measurement used to assess students’ IQ level.
- (iii) The Digit Span Test, forward and backward, which is a subtest of Malin's Intelligence Test for Indian Children^29^, was conducted to assess students’ working memory status.
- (iv) The Bender Gestalt Test was administered to assess visual-motor functioning, developmental disorders and neurological impairments in students^30^.

### Electrophysiological measurements

qEEGs were recorded on the participants before and after the intervention was implemented. The gap between the pre and post-EEG assessments was two months. They were recorded in the psychophysiology laboratory of the Department of Applied Psychology, University of Delhi, after school hours. EEGs were acquired under two conditions: two minutes of resting with the eyes closed (with meditation music) and two minutes with the eyes open (during which the student were asked to focus at one dot on a screen). 

Data were acquired through the BIOPAC system MP36 (BIOPAC Systems, Inc.), from four digital channels in the 10-20 system, and were analyzed using the AcqKnowledge 4.1 software MP150, referenced to the linked earlobes (A1-A2). The reference for the EEG bandwidth of the waveforms was placed within the range of 0.5 Hz to 70 Hz. The input impedance of all electrodes was set at 2 MΩ, and the output impedance was set at 50 Ω, with a gain of 50,000. 

EEG data were sampled at the rate of 2000 samples/second, for every 0.5 ms. These raw EEG signals were processed with the aid of the AcqKnowledge software. The delta (0.5-4.0 Hz), theta (4-8 Hz), alpha (8-13 Hz), beta (13-30 Hz) and gamma (30-60 Hz) frequency bands were then extracted from the signals. The average absolute and relative powers of the frequency bands for the 50 participants were calculated in eyes-closed and eyes-open states. 

**Figure 1.**
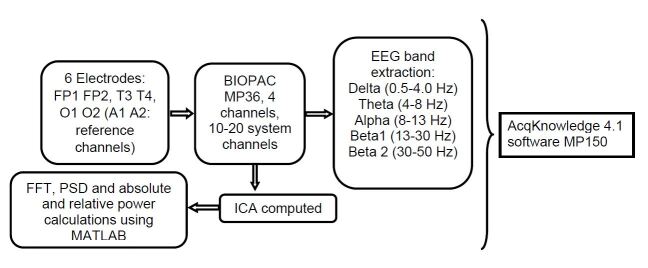
Representation of EEG extraction and power calculation.

The EEGs had been recorded with six electrodes that were placed on the scalp in accordance with the international 10-20 system^31^. The electrodes selected for the experiments were FP1, FP2, T3, T4, O1 and O2. A1 and A2 were taken as the ground electrodes. Independent component analysis (ICA) was done. Later, the fast Fourier transform (FFT), power spectrum density (PSD), absolute band power, and relative band power of the EEG signals were calculated using MATLAB coding^32^. Figure 1shows a schematic diagram for EEG extraction and power computing.

### Experimental hypothesis

In several studies, the most common qEEG pattern among children with learning disorders, recorded during a resting state (eyes closed), was revealed to consist of an excess of slow theta activity and a deficit of alpha activityt^12^^,^^33^, compared with normal children. Among cases of severe disability, excess delta activity was also observed^12^. For children with verbal disabilities and children with LDNOS, differences in EEG oscillations at delta, theta, alpha and beta-1 frequencies under eyes-open conditions were reported in the left temporal cortex. In the frontal region, a higher theta/alpha ratio among children with LDNOS was also reported^34^. In another study, children with learning disabilities presented greater absolute delta and theta powers than those of normal children. Several findings have suggested increased delta, theta and alpha1 powers in children with learning disabilities, due to the immaturity of brain development.

A highly positive correlation between the relative alpha2 power and IQ quotients implies that the alpha2 band power is associated with greater maturity of the brain's electrical activity. On the other hand, the frequency of the alpha1 fraction has a negative correlation with IQ, which signifies lower brain maturity^22^. EEGs may provide corroboration for the biological brain characteristics involved in children with learning disabilities; brain immaturity may be one of the possible factors that originate such disabilities. 

Additionally, several studies have reported the presence of EEG abnormalities in children with learning problems, i.e. slow delta and theta activity in the frontal and temporal regions^35^^,^^36^. Mann et al.^37^ reported that children with hyperactivity, impulsivity and attention-related problems had higher absolute theta activity in the frontal regions during cognitive tasks and decreased beta activity in the posterior and temporal regions during tasks that required sustained attention. Beta activity (15-30 Hz) has been recognized to play an essential role in cognitive activity. Improving beta and alpha activity while reducing theta activity can help improve attention and behavioral control and diminish hyperactivity and impulsivity among children with learning problems^38^^,^^39^.

Given that significant heterogeneity exists among subjects with learning problems ^40^ and considering the significant findings from previous research studies, we considered it necessary to explore EEG characterizations of children with difficulty in learning under both eyes-closed and eyes-open conditions after the intervention had been presented. Two hypotheses merited immediate attention: H1: PEABLS interventions among children with learning difficulty would increase slow alpha and beta band power values and reduce theta band power values at the FP1, FP2, T3, T4, O1 and O2 scalp locations; and H2: There would be a significant relationship between EEG band power values and scores from cognitive measurements.

### Data analysis

Cognitive and electrophysiological variables assessed at baseline and post-intervention were compared. EEG recordings were made while the subjects were awake and resting. The values for absolute and relative band powers were calculated from the pre and post-intervention EEG results. The delta, theta, alpha, and beta bands of the EEGs were compared using the t test for paired samples. Correlations between EEG parameters and cognitive data (obtained from IQ scores in RCPM), BGT and working memory status (obtained from the Digit Span Test, forward and backward) were also studied, using Pearson's correlation analysis.

Paired t tests were performed to compare the pre and post-assessments of the absolute power (AP) and relative power (RP) of EEG waveforms.

## RESULTS

Visual inspection of histograms, normal Q-Q plots and box plots showed that the ages and grades of the participants were approximately normally distributed, with skewness of -0.210 (SE = 0.337) and kurtosis of -1.165 (SE = 0.662) for the age variable and skewness of 0.151 (SE = 0.337) and kurtosis of -1.106 (SE = 0.662) for the grade variable. Our data for both the age and the grade variables were somewhat skewed and kurtotic, while the other data were approximately normally distributed in terms of skewness and kurtosis^41^^,^^42^. 

Under the eyes-closed condition (Table 2), there were significant decreases in the mean scores for the final Beta and Gamma band power values in the occipital region after the intervention. On the other hand, under the eyes-open condition (Table 3), significant depletion in absolute alpha band power values at the temporal region was observed. Significant decreases in absolute beta, delta and gamma band power values were also observed.

**Table 2.** Absolute power values for beta and gamma at the O1 O2 locations for the group of students with learning difficulty before (pre) and after (post) intervention and the p values for the respective comparisons in task 1 (eyes closed).

Absolute power values	O1 O2			
	Pre-intervention	Post-intervention	t value	p
Beta	8.01 (15.41)	2.45 (1.87)	2.57	0.01**
Gamma	0.68 (1.38)	0.14 (.16)	2.73	0.00**

**p < 0.01.

**Table 3.** Absolute alpha, beta, theta, delta and gamma values at the FP1 FP2 and T3 T4 locations for the group of students with learning difficulty before (pre) and after (post) intervention and the p values for the respective comparisons in task 2 (eyes open).

Absolute power values	FP1 FP2				T3 T4			
N = 50	Pre-intervention	Post-intervention	t value	p	Pre-intervention	Post-intervention	t value	p
Alpha	2.39 (2.34)	2.2 (1.31)	0.52	0.60	6.01 (4.05)	4.86 (3.98)	1.92	0.05*
Beta	9.09 (12.13)	6.05 (6.41)	1.95	0.05*	14.08 (8.9)	8.27 (5.4)	4.06	0.00**
Delta	180.22 (508.02)	48.86 (29.18)	1.92	0.05*	198.48 (565.85)	61.03 (77.94)	1.9	0.05*
Gamma	0.59 (1.63)	0.26 (0.31)	1.498	0.14	0.68 (0.63)	0.41 (0.41)	2.58	0.01**

df: 49; *p < 0.05; **p < 0.01.

Relative EEG band power values were also calculated. Under eyes-closed conditions (Table 4), the relative alpha and delta band power values in the prefrontal region and the relative gamma band power values in the temporal region significantly increased after the intervention. In contrast, significant declines in relative band power values for alpha, beta and gamma were observed in the occipital region. The relative beta band power values at the temporal scalp location also decreased post-intervention. 

**Table 4.** Relative alpha, beta, theta, delta and gamma values at the FP1 FP2, T3 T4 and O1 O2 locations for the group of students with learning difficulty before (pre) and after (post) intervention and the p values for the respective comparisons in task 1 (eyes closed).

Relative power values	FP1 FP2				T3 T4				O1 O2			
N = 50	Pre-intervention	Post-intervention	t value	P	Pre-intervention	Post-intervention	t value	p	Pre-intervention	Post-intervention	t value	p
Alpha	0.38 (0.05)	0.4 (0.05)	2.01	0.03*	0.51 (0.12)	0.51 (0.11)	0.008	0.99	0.48 (0.65)	0.31 0(.06)	8.25	0.00**
Beta	0.75 (0.07)	0.73 (0.06)	1.52	0.13	0.65 (0.12)	0.6 (0.12)	2.65	0.01**	0.66 (0.15)	0.57 (0.17)	3.38	0.00**
Delta	0.82 (0.1)	0.86 (0.07)	2.26	0.02*	0.81 (0.12)	0.84 (0.1)	0.88	0.37	0.74 (0.17)	0.78 (0.15)	1.132	0.26
Gamma	0.17 (0.07)	0.19 (0.07)	1.57	0.12	0.19 (0.06)	0.22 (0.1)	2.01	0.05*	0.27 (0.07)	0.23 (0.09)	2.45	0.01**

df: 49; *p < 0.05; **p < 0.01.

Under eyes-open conditions (Table 5), after the intervention, there were significant declines in relative band power values for beta in the prefrontal and temporal region and for theta in the occipital region. There were significant increases in post-intervention relative band power values for alpha, theta and delta in the prefrontal region. The relative band power values for alpha and theta in the temporal region and for gamma in the occipital region significantly increased. 

**Table 5.** Relative alpha, beta, theta, delta and gamma values at the FP1 FP2, T3 T4 and O1 O2 locations for the group of students with learning difficulty before (pre) and after (post) intervention and the p values for the respective comparisons in task 2 (eyes open).

Relative power values	FP1 FP2				T3 T4				O1 O2			
N = 50	Pre-intervention	Post-intervention	t value	p	Pre-intervention	Post-intervention	t value	p	Pre-intervention	Post-intervention	t value	p
Alpha	0.34 (0.07)	0.37 (0.05)	2.85	0.00**	0.40 (0.1)	0.44 (0.09)	2.59	0.01**	0.35 (0.08)	0.35 (0.08)	0.19	0.84
Beta	0.76 (0.08)	0.74 (0.07)	1.94	0.05*	0.73 (0.08)	0.7 (0.08)	2.43	0.01**	0.77 (0.08)	0.78 (0.05)	1.38	0.17
Theta	0.5 (0.05)	0.54 (0.04)	4.75	0.00**	0.52 (0.06)	0.54 (0.06)	2.53	0.01**	0.43 (0.07)	0.4 (0.09)	1.99	0.05*
Delta	0.88 (0.06)	0.91 (0.02)	2.79	0.00**	0.85 (0.13)	0.88 (0.03)	1.45	0.15	0.86 (0.05)	0.87 (0.03)	1.56	0.12
Gamma	0.2 (0.06)	0.19 (0.06)	0.72	0.47	0.22 (0.05)	0.23 (0.07)	0.51	0.61	0.31 (0.06)	0.33 (0.05)	2.75	0.00**

df: 49; *p < 0.05, **p < 0.01.

Pearson's correlation coefficient was determined between relative EEG band power values on scalp locations (FP1, FP2; T3, T4; and O1, O2) and IQ, working memory and BGT. Table 6 depicts the correlation coefficients for relative EEG band powers under eyes-closed and eyes-open conditions in relation to cognitive measurements. The relative band power value for alpha in the occipital region was significantly correlated with working memory in the digit span test (forward and backward) and with BGT. In the temporal and occipital regions, the relative band power values for beta and theta were significantly correlated with the IQ score from the RCPM and with working memory in the digit span test (forward and backward). The relative band power for beta was significantly correlated with IQ score from the RCPM and with working memory in the digit span test (forward). The relative band power value for gamma in the prefrontal region was significantly correlated with the BGT score.

**Table 6.** Pearson’s correlation coefficients for the relative alpha, beta, theta, delta and gamma values at the FP1 FP2, T3 T4 and O1 O2 locations for the group of students with learning difficulty in relation to the IQ from Raven’s Color Progressive Matrices (RCPM), working memory from the Digit Span Test (forward and backward) and Bender Gestalt Test (BGT), under eyes-closed and eyes-open conditions.

Location	IQ		Working memory				BGT	
			Memory (F)		Memory (B)			
	Eyes closed	Eyes open	Eyes closed	Eyes open	Eyes closed	Eyes open	Eyes closed	Eyes open
FP1 FP2 Alpha	0.12	0.11	0.07	-0.10	0.19	-0.11	0.02	0.25*
O1 O2 Alpha	0.11	0.03	0.36^*^	0.05	0.32^*^	0.10	-0.34^*^	-0.10
FP1 FP2 Beta	-0.39^**^	-0.23*	-0.22*	-0.22*	-0.23*	-0.08	-0.03	-0.13
T3 T4 Beta	-0.23*	-0.19*	-0.24*	-0.24*	-0.11	0.10	-0.06	-0.04
O1 O2 Beta	-0.25*	-0.07	-0.15	-0.27*	-0.08	0.05	0.01	0.14
FP1 FP2 Theta	0.20*	0.04	0.32^*^	0.15	0.18	-0.05	-0.19*	0.04
T3 T4 Theta	-0.03	0.07	-0.23*	-0.10	-0.14	-0.11	0.06	0.10
O1 O2 Theta	-0.12	-0.14	-0.08	0.04	-0.19	-0.28^*^	0.02	-0.07
FP1 FP2 Delta	0.14	0.07	-0.05	0.07	0.12	0.32^*^	0.06	0.02
T3 T4 Delta	-0.06	-0.23*	-0.07	0.11	0.13	0.19*	0.10	0.06
FP1 FP2 Gamma	0.13	0.05	0.02	0.12	0.14	0.15	-0.10	-0.19*

IQ: measured from Ravens’ Color Progressive Matrices; *p < 0.05, **p < 0.01.

## DISCUSSION

The aim of the present study was to evaluate electroencephalographic activity under eyes-closed and eyes-open conditions before and after introducing PEABLS among students with learning difficulty. PEABLS has been shown to yield significant improvements in academic, cognitive and behavioral measurements^26^^,^^43^. In the present study too, after introduction of PEABLS to students with difficulty in learning, significant improvements in the absolute and relative power values of waveforms at the FP1 FP2, T3 T4 and O1 O2 scalp locations were achieved. 

Some of the significant findings included increased relative alpha power values at the FP1 FP2 and T3 T4 scalp locations under eyes-closed and eyes-open conditions. There were also significant increases in relative theta and delta power values at the FP1 FP2 and T3 T4 scalp locations under eyes-open conditions. The relative beta band power values significantly decreased at the T3 T4 and O1 O2 scalp locations under eyes-closed conditions.

Furthermore, the BGT scores were significantly negatively correlated with O1 O2 alpha and FP1 FP2 theta under eyes-closed conditions. In contrast, BGT scores were significantly positively correlated with FP1 FP2 alpha and negatively correlated with FP1 FP2 gamma. Memory scores were positively correlated with O1 O2 alpha under eyes-closed conditions. Both IQ and memory scores were negatively correlated with beta power values at the FP1 FP2, T3 T4 and O1 O2 locations under eyes-closed and eyes-open conditions. Another important finding was that IQ was positively correlated with theta power values at the FP1 FP2 locations under eyes-closed conditions. 

The findings indicated that at the baseline assessment, high absolute delta and theta powers were observed. The absolute alpha power was low. Similar findings were indicated in other studies in which students with learning disability were compared with normal participants^9^^,^^10^^,^^12^^,^^22^. After the PEABLS intervention had been undertaken, significant decreases in absolute Beta and Gamma band power values in the occipital region were noticed under eyes-closed conditions. On the other hand, under eyes-open conditions, diminution of absolute alpha band power values was observed in the temporal region, and of absolute beta, delta and gamma band power values. In the present study and one other study^14^, decreased activity in occipital regions was possibly due to the maturity effect: the brain electrical activity in these regions occurred much faster than what was referred to as slow activities. 

The relative alpha and delta powers in the prefrontal region and the relative gamma power in the temporal region significantly increased after the intervention, as did the relative alpha, beta and gamma powers in the occipital region. Studies comparing EEG results from before to after the intervention are scarce. However, some other studies^21^^,^^22^revealed that increased delta and alpha powers in children with learning disabilities were related to the immaturity of brain development, which decreases with age.

Relative beta was also low at the temporal scalp location after the intervention under eyes-closed conditions. There was a decline in relative beta power under eyes-open conditions in the prefrontal and temporal region and in relative theta power in the occipital region, and increased relative alpha, which suggests that the brain was relaxed and active. Alpha and theta in the temporal region, and relative gamma in the occipital region, significantly increased after the intervention, which was suggestive of a calm, relaxed and non-stressed brain state. 

The relative alpha power in the occipital region was significantly correlated with working memory in the digit span test (forward and backward) and in BGT. These correlations show the association of alpha power with the maturity of the brain's electrical activity^44^. The relative beta and theta were significantly correlated with the IQ score in the RCPM and with working memory in the digit span test (forward and backward) in the temporal and occipital regions. The relative beta was significantly correlated with the IQ score in the RCPM and with working memory in the digit span test (forward). The relative gamma in the prefrontal region was significantly correlated with the BGT score. 

This study attempted to identify EEG activity changes after PEABLS, an intervention that follows a cognitive-behavioral approach. The duration of the intervention was two months (16 sessions). Significant changes in absolute and relative band power values were noticed in the prefrontal, temporal and occipital scalp locations bilaterally. However, the desired results, such as increases in alpha, beta and gamma band power values and decreases in theta and delta band power values, which improve children's cognitive abilities, remain to be achieved. Nonetheless, cognitive and behavioral improvements were noticed post-intervention. Multiple morbidity conditions and environmental conditions may affect brain activity, to show significant changes of short duration^45^.

The duration of the intervention can be increased, and intermittent EEG recordings can be made at regular intervals to check whether the intervention is enabling a move towards the desired path. The importance of these findings cannot be overstated, given that appropriate early assessments and interventions may help change developmental trajectories and long-term outcomes. Future research should explore early screenings and potential interventions to help maximize children's brain development and cognitive potential.

Despite the best efforts made to control the variables under study, the present study had some limitations. Nevertheless, the PEABLS did bring considerable enhancement of self-regulated behavior, thereby promoting the students’ resilience and improving their academic skills. Due to lack of time and resources, the baseline and post-intervention assessments with EEG were only possible for the students who participated in the experimental group and not for the control group. Therefore, experimental group and control group comparison after the intervention was not possible. Moreover, variations among these students in the age group from 8 to 12 years (corresponding to the education levels of 3^rd^ to 7^th^ grade) may have caused heterogeneity because neurodevelopment during this age range is accelerated, thereby modulating the learning process. The maturation effect may have significantly influenced the results. It can be suggested that future research should be conducted at a single age or using a single classification of biological maturation, in order to maintain sample homogeneity.
